# Experiences and perceptions of receiving and prescribing rehabilitation in adults with cystic fibrosis undergoing lung transplantation

**DOI:** 10.1177/14799731221139293

**Published:** 2023-03-29

**Authors:** Lisa Wickerson, Rajan Grewal, Lianne G Singer, Cecilia Chaparro

**Affiliations:** 1 Toronto Lung Transplant Program, 7989 University Health Network, Toronto, ON, Canada; 2 Department of Physical Therapy, 7938 University of Toronto, Toronto, ON, Canada; 3 Department of Medicine, 7938 University of Toronto, Toronto, ON, Canada

**Keywords:** Cystic fibrosis, rehabilitation, tele-rehabilitation, lung transplantation

## Abstract

**Background:**

Rehabilitation is prescribed to optimize fitness before lung transplantation (LTx) and facilitate post-transplant recovery. Individuals with cystic fibrosis (CF) may experience unique health issues that impact participation.

**Methods:**

Patient and healthcare provider semi-structured interviews were administered to explore perceptions and experiences of rehabilitation before and after LTx in adults with CF. Interviews were analyzed via inductive thematic analysis.

**Results:**

Eleven participants were interviewed between February and October 2021 (five patients, median 28 (IQR 27–29) years, one awaiting re-LTx, four following first or second LTx) and six healthcare providers. Rehabilitation was delivered both in-person and virtually using a remote monitoring App. Six key themes emerged: (i) structured exercise benefits both physical and mental health, (ii) CF-specific physiological impairments were a large barrier, (iii) supportive in-person or virtual relationships facilitated participation, (iv) CF-specific evidence and resources are needed, (v) tele-rehabilitation experiences during the COVID-19 pandemic resulted in preferences for a hybrid model and (vi) virtual platforms and clinical workflows require further optimization. There was good engagement with remote data entry alongside satisfaction with virtual support.

**Conclusions:**

Structured rehabilitation provided multiple benefits and a hybrid model was preferred going forward. Future optimization of tele-rehabilitation processes and increased evidence to support exercise along the continuum of CF care are needed.

## Introduction

Cystic fibrosis (CF) is a multisystem disease, primarily characterized by progressive lung disease leading to respiratory failure or referral to LTx.^1,2^ Skeletal muscle impairments, reduced exercise capacity and lower levels of physical activity are also present.^3–6^ There is evidence to support exercise in improving exercise capacity, pulmonary function, health-related quality and mucociliary clearance in CF.^7,8^ Higher exercise capacity in LTx candidates is associated with reduced morbidity and mortality both pre- and post transplant,^9–11^ and rehabilitation, both in the inpatient and outpatient settings is a recommended component of a LTx program.^12^

Tele-rehabilitation (rehabilitation delivered over telecommunication networks and the internet) has the potential to support patients closer to home and increase access and adherence to rehabilitation. This may be particularly relevant to the CF population who are at risk of cross-colonization and infection, and have a high daily regimen of medications, nutritional management and airway clearance therapies.^13^ Hospitalizations for respiratory exacerbations are common pre-LTx and can disrupt the rehabilitation program. Prior to the COVID-19 pandemic, on-site supervised rehabilitation was standard of care before and after LTx, and less was known of tele-rehabilitation in this population. Web and App-based exercise programs have been reported in three pilot studies of LTx candidates and recipients.^14–16^ One of these studies focused solely on CF LTx candidates and found that participants who used a commercially available fitness app with asynchronous monitoring and communication completed more exercise sessions over 12 weeks than the hospital-based exercise group, although there was a high variability of completed sessions.^16^ A program evaluation performed at our LTx centre following the implementation of tele-rehabilitation in 2020 in response to the COVID-19 pandemic reported good engagement despite barriers of exercise equipment, however this evaluation was not specific to LTx candidates or recipients with CF.^17^

This study involved semi-structured interviews with patients and healthcare providers. The aim was to gain an in-depth understanding of the experiences and perceptions of receiving and prescribing rehabilitation care pre-LTx and in the early post-LTx period in adults with CF. Patient usage and satisfaction data from a remote monitoring App that was being utilized to support clinical outpatient care was also collected.

## Materials and methods

### Study design

This study was conducted at a single Canadian LTx center (University Health Network, Toronto, Canada). Ethics approval was obtained from the research ethics board (REB #20-6015). All participants provided written informed consent that was collected remotely.

### Rehabilitation delivery

For outpatients, rehabilitation during March 2020-June 2021 was delivered primarily at home with in-person sessions occurring between once a week or once a month depending on covid-19 on-site restrictions and patient stability, conditioning level, access to home exercise equipment and adherence to home exercise. There was no patient fee for in-person rehabilitation or for use of the remote monitoring App. For inpatients, in-person rehabilitation occurred at the adult CF centre pre-transplant and the transplant centre post-transplant.

### Sampling and recruitment

A purposive sampling technique was utilized. Eligible participants included two groups: (i) patients aged 18 years of age or older with CF who were waiting for lung transplant (initial or re-transplant) or who underwent a lung transplant between March 2020 and June 2021 and (ii) healthcare providers at the University Health Network or its’ affiliated adult CF center (Unity Health Toronto) who delivered and/or made clinical decisions around lung transplant rehabilitation.

### Informed consent process

#### Patients

The study lead who was also a clinician in the lung transplant program identified eligible participants. A clinician in the circle of care and not on the research team approached the patient either in-person during on-site rehabilitation or during a virtual rehabilitation visit to ask if they were willing to be contacted to learn about the study. The research assistant then called the patient to ask for permission to use their email to send the informed consent form by Institutional File Share and plan a time to call them back to discuss. Two FileShare passwords were shared verbally by phone in order to download and unzip the file. The consent process occurred through Microsoft Teams or by phone. The patient signed and returned the consent form in person, by email or via FileShare.

#### Providers

Providers were contacted either in-person or by email by the study lead who was a clinical colleague to tell them about the study. If interested, the research assistant sent an email with an attached consent form. The consent process occurred through Microsoft Teams or by phone. The provider signed and returned the consent form in person or by email.

### Data collection procedures

#### Semi-structured virtual one-on-one interviews

Two interview guides (patient and provider) were developed based on a literature review, expert opinion and previous surveys that one researcher (LW) had used previously to explore perceptions of e-health interventions in solid organ transplant recipients.^18^ Supplement 1S One researcher (RG) conducted the interviews in English between February and October 2021 using Microsoft Teams.

#### Patient usage and satisfaction data from the remote care monitoring App

An online, remote care monitoring App that was being used for clinical care since March 2020 walked people through the exercise program and allowed manual entry of the amount of exercise performed at home (e.g. frequency, intensity, time and type) and pre- and post-exercise biometrics (e.g. oxygen saturation, heart rate, Borg dyspnea and leg fatigue scores). Satisfaction surveys were also sent in the App to LTx candidates after one month of using the App and to LTx recipients three months after transplant. Supplement 2S The remote care monitoring App has been described in detail elsewhere.^17^

### Thematic analysis

Interviews were recorded on an external digital recorder and later de-identified and transcribed verbatim. Transcripts were entered into NVIVO 12 Plus data management software. One researcher (LW) read through all transcripts and generated initial codes using an iterative-inductive analytical strategy which were organized into a coding framework. A second researcher (RG) independently applied this coding framework to one patient and one provider transcript. A meeting was held to discuss the codes and agree upon terminology which were further refined. LW re-applied this new coding framework to all transcripts and identified broad themes. Key themes were reviewed, refined and named. Both patient and provider codes were combined into the same theme were relevant, otherwise a separate theme was created.

## Results

Between February and October 2021 11 people were interviewed including five patients (4 women, median age 28 (IQR 27-29) years, one awaiting re-LTx, four following first or second LTx) and six healthcare professionals (4 physiotherapists, 1 physiotherapy assistant, 1 respirologist). Participant characteristics are described in Table 1. During this time frame there were three waves of COVID-19 accompanied with varying levels of on-site ambulatory and community restrictions.^19^ Patient engagement using the remote monitoring app was high with entry of home-based exercise pre- and post LTx. Table 2 All five patient participants agreed or strongly agreed that the remote patient monitoring App supported their journey in preparing for and/or recovering from surgery, empowered them to manage their health condition and provided support from their healthcare team. Six key themes were identified regarding the experience and perceptions of receiving and prescribing rehabilitation care pre- and post lung transplantation for CF. Themes along with illustrative quotes are presented in Table 3.

**Table 1.** Participant characteristics (n = 11).

Characteristic	median (IQR), [range] or n (%)
Patient participants (n = 5)	
Female gender	4 (80)
Age (years)	28 (27–29)
Pre-LTx (re-transplant)	1 (20)
Post-LTx (1^st^ transplant)	2 (40)
Post-LTx (re-transplant)	2 (40)
Time since initial LTx (months)	[1–346]
Time since re-LTx	[6–7]
Pre LTx	
6MWD (m)	402 (374–428)
6MWD (% pred)	49 (45–53)
FEV_1_ (L)	0.7 (0.6–1.5)
FEV_1_ (% pred)	18 (18–45)
BMI (kg/m^2^)	18 (16–22)
Healthcare Provider participants (n = 6)	
Female gender	4 (66)
Occupation
PT	4 (66)
PTA	1 (17)
MD	1 (17)
Practice setting	
Inpatient	2 (33.3)
Outpatient	2 (33.3)
Inpatient and outpatient	2 (33.3)
Institution	
LTx center	3 (50%)
Adult CF center	3 (50%)

LTx: lung transplantation; 6MWD; six-minute walk distance; FEV1: forced vital capacity in one second; BMI: body mass index; PT: physiotherapist; PTA: physiotherapy assistant; MD: medical doctor.

Eligible/invited participants: The three physiotherapists from the adult CF centre as well as three physiotherapist assistants and three physiotherapists from the lung transplant centre who deliver outpatient rehabilitation and one physician specializing in lung transplant rehabilitation were eligible. One of the physiotherapists from the lung transplant centre was leading the research study so was therefore not invited resulting in 9 potential participants.

There were 19 eligible patients. Six did not attend an in-person or a virtual rehabilitation visit during the study timeframe and therefore were not approached. 13 were approached and of these 7 did not response to or complete the virtual consent process, 1 died before consent with the remaining 5 included in the study.

**Table 2.** Home-based exercise recorded in the remote monitoring App pre- and post-transplantation.^a^

	Pre-transplant	Post-transplant (first 3 months)
LTx candidate re-Tx	Walk-20–60 mins^b^	N/A
	Cycle- 20–25 mins	
	Upper extremity weights^c^ - 3–5 lbs	
	Lower extremity weights – 3 lbs	
	Oxygen use: 6L NP	
LTx recipient 1^st^ Tx	Walk- 4000–6000 steps,	Walk – 30–60 mins
	- treadmill 2.2 mph, 20–25 mins	Cycle – 20 mins
	Cycle -10–20 mins	Upper extremity weights- 4 lbs
	Upper extremity weights- 3–5 lbs	Lower extremity weights – 2–3 lbs
	Lower extremity weights- 3 lbs	
	Oxygen use: none	
LTx recipient 1^st^ Tx	Walk - 1600–2700 steps,	Walk - 2628-7264 steps,
	- treadmill 1.6–1.7 mph, 20 mins	- treadmill 2.2-2.3 mph, 20 mins
	Upper extremity weights -3 lbs	Cycle -10-15 mins
	Lower extremity weights – no weight	Upper extremity weights -3 lbs
	Oxygen use: 8L NP	Lower extremity weights – no weight
LTx recipient re-Tx	Walk – 50 m	Walk – 1 Km
	Cycle – 20–60 mins	Cycle -10–30 minutes
	Upper extremity weights – 3 lbs	Upper extremity – 3 lbs
		Lower extremity weights- 0–3 lbs
	Lower extremity weights – no weight	
	Oxygen use: 4 L NP	
LTx recipient re-Tx	Admitted to the intensive care unit and bridged to transplant on vv-ecmo	Admitted to inpatient rehabilitation

LTx: lung transplantation, re-Tx: re-transplantation, NP: nasal prongs, vv-ecmo: venovenous extracoroporeal membrane oxygenation.

^a^minimum home-based exercise requirement and entry of 3 days a week.

^b^shows exercise progression over time.

^c^weight exercise prescribed as 1 to 3 sets of 10 reps.

**Table 3.** Themes and quotes illustrating perceptions of lung transplant rehabilitation.

Themes illustrative quotes	
Structured exercise benefits both physical and mental health pre- and post-transplant	***Physical health***
	*Breathing has become a bit easier since doing the exercise.* [Patient 3]
	*Rehab prior to transplant is important to maintain or even advance muscle mass because it’s a big operation, and the recovery is just as crucial.* [Patient 5]
	*I’ve worked with patients with 13% lung function and from an exercise perspective even people with tiny, tiny bodies are capable of doing an awful lot in terms of exercise. So better outcomes post transplant and faster recovery.* [Provider 1]
	*It’s a big drain on the system to have the operation and we want to help them get as strong as possible before so that they can get up and get walking, get out of the hospital then move forward and move on with their life.* [Provider 6]
	***Mental health***
	*Um, my favourite thing about it was the structure, obviously. Something to keep in the back of my mind whenever I was feeling particularly sick, that I still do 3 times a week you know, go on the treadmill, use my little weights. It gives you some kind of structure and positivity. I’ve done something towards a goal.* [Patient 2]
	*I could actually get up and do stuff on my own -made me feel better mentally because for so long you had to have people do stuff for you.* [Patient 4]
	*It’s reassuring that I am doing the best I can for the lungs that I have. That’s a way of honouring my donor too. I want to do everything I can do keep myself healthy, not only for myself but for them. I have a big life that I want to lead. Exercise gives me time to just focus on my breathing, kind of inhale the good things and exhale the bad things. Having the exercise routine has greatly impacted the positive mental stamina, essentially.* [Patient 5]
	*It makes me feel better mentally and physically, because it's just that boost of energy to keep you going throughout the day. It tires you out but at the same time it makes you feel better. If I just sit on the couch and do nothing, I'll just wanna do nothing all day, and I feel like crap basically.* [Patient 4]
CF-specific physiological impairments were a large barrier to exercise	*Of course shortness of breath is kind of a huge thing for anyone with lung disease but with CF it’s so much more than that, your body’s working so hard to breathe because the mucus in your lungs is so sticky. There’s more degrading in your body than just your lungs, your digestive system’s working overtime as well so I think it's just harder in terms of keeping your energy up*. [Patient 2]
	*So the issue with CF, if there anything like me and my brother it's that we cough up blood and then we can't really exercise after, we have to be careful.* [Patient 4]
	*I had a number of big exacerbations that really put a damper on my ability to exercise especially when I was in hospital.* [Patient 2]
	*Lower BMI, lower muscle mass, issues with bone density, issues with postural kyphosis, and problems with urinary incontinence which obviously stems from lots of coughing and also from weaker muscles including the pelvic floor*. [Provider 1]
	*I would say fatigue is a common treatment burden probably more with CF compared to other patients, and CF patients also have other co-morbidities like DIOS. CF is more multisystem.* [Provider 2]
	*People with CF need to eat double, triple the amount of calories you and I do just to survive, and they’re still malnourished and they’re still underweight. And again, people get worried, OK I’m going to exercise now – is that going to end up burning the calories that I just spent all morning trying to swallow, even though I feel kinda permanently nauseous and don’t want to have to do it?* [Provider 1]
	***Treatment burden***
	*I just wake up and I take my pills and stuff and I just I don’t know I’ve gotten used to it, it’s my life so I don't think that’s a barrier.* [Patient 2]
	*We’ve been doing this our whole life. So, medications, manual percussion therapy, PEP mask, it’s something we’re used to. It’s not really a barrier, if anything the physio rehab is like a supplement to what we’re doing. Um, for other illnesses that have brought transplant on, I’m sure that especially later diagnosis, they’re put on so many different meds and that in itself is overwhelming and then throw in having to do the rehab program as well could definitely be a barrier. But in terms of CF I think it’s just so normal to us that adding on the physio rehab is just one other thing, you just do it, you do what you can, you do your best, you get on with it*. [Patient 5]
	***Infection control***
	*So, I know for me, I had a great deal of hesitancy, um, looking for a gym membership. I could if I wanted to, go to a gym and I would be wiping everything down before I touch it. For me, I’m comfortable doing it at home.* [Patient 5]
	*Infection can be a* [CF-specific] *barrier but not so much now with COVID because life is really different. But pre-COVID we’d have to have a separate schedule for CF. You can come in at this time and go in this door, this side of the room, don’t come in that door, come over here, let me clean all that.* [Provider 6]
Collaborative and supportive relationships, either in-person or virtual, facilitated exercise participation	*I like the consultation with the physiotherapists and I like the fact that they actually push you to do it. It is a little bit more motivating when there is someone standing over you saying OK now you gotta do this.* [Patient 1]
	*I have patients who worried about doing exercise because they worry that would actually decrease their weight, so we work with the dietician to make sure that they’re eating enough to compensate.* [Provider 5]
	*Working 1 on 1 with somebody really getting to know them, developing that relationship, and then coming up with kind of collaborative goals that you set together and then just kind of checking in with them.* [Provider 1]
	*We communicate with our psychiatry team and they assist with any anti-anxiety medication, that when we do see them they can do a bit more with us.* [Provider 4]
	*I really liked that* [physios] *could tell when you were struggling almost before you could even tell yourself. They say like “You gotta either dial it back a little bit,” or in some cases patients are too scared to do, more and they’ll say “Okay, let’s try the next step here.” So, I found that they’re very in tune with the patients.* [Patient 5]
	*My husband asks me if I did my workout today and joins me every once in awhile.* [Patient 3]
	*I have a competition sometimes with my friends because we have Fitbits, so we compete to see who can take the most amount of steps on a weekend.* [Patient 4]
More CF-specific exercise evidence, guidance and resources are needed	*Now what we’re seeing is people post CFTR modulator who start taking this drug very deconditioned, then they start improving their lung function, they get they don’t get sick as often, they put on 15 or 20 pounds in body weight. All of a sudden, they realize holy moly I can’t do any of this stuff, not because of my lungs, but because I’m so de-conditioned that I haven’t exercised.* [Provider 1]
	*The clinic that I grew up going to, they never gave out a pamphlet on exercises to do. I remember as a kid, they promoted exercise certainly, but they never gave my parents any real guidance on exactly what exercises are best. Some instruction as to the benefits of exercise that will help expand your rib cage, get you breathing deeper could be beneficial on the pediatric side, so starting out early.* [Patient 5]
	*Beam CF platform is basically just exercise focused, so it has on demand classes, and live classes as well everyday for a variety of fitness levels, so I’ve recently started directing our patients to that. It’s a beautiful website, it is privately owned so there is a cost associated with it, but the way I look at it is that often when patients make a financial commitment to exercise they’re more likely to participate in it. It’s all people either with CF or physios that are working with CF.* [Provider 1]
	*The walking or running have more bouncing effect and it actually helps them improve more sputum out of the airway. Now quite a bit of debate on whether they can actually use exercise as a way to airway clearance.* [Provider 5]
Experience with tele-rehabilitation and eHealth tools during the COVID-19 pandemic resulted in preferences for a hybrid model	***Patient perspectives***
	*I think that the rigidity of 3 times a week in-person could probably be loosened. I agree for a group of patients it’s definitely beneficial to be there 3 times a week and having that input from the team but I do think with apps there are a lot of people who can have a little bit more fluidity to their schedules. Being able to just go downstairs and use my treadmill and weights was much better for me. So I think it needs to be more case-by-case and less of a broad mandate.* [Patient 2]
	*I think a mix, if not more so in person. At least for me, I felt like the in-person sessions were more beneficial. I felt more confident knowing that somebody was there to oversee things. I do find that the in-person is a better use of your energy and your time, but I think it could be optional for you to do it at home. If you really feel like you can do it, you should be allowed to. But if they lack confidence in your ability, I think they should maybe scale it more towards in-person.* [Patient 5]
	*I think the mix, hybrid worked for me. I was fortunate that I had my wife here and I’m fairly motivated myself to continue with the exercises at home, some people may not be though, so more in-person visits might be better.* [Patient 1]
	*[the physiotherapist] was fantastic to reach out to. She is always on time and prompt with her responses. Every 2 weeks she checked in with me on the App, had a phone conversation with me or video whatever we could fit in, and was willing to make adjustments or do whatever needed to be done.* [Patient 2]
	*As for the App, I think it’s fantastic. In terms of usage, it has all of the reminders you need. It has the exercise program that they use, what to do when you get the call for the transplant, it talks about recovery, about maintaining health. It’s everything that they had in those giant books that they used to give out in an App, which I know for at least the younger generation it’s great.* [Patient 5]
	***Provider perspectives***
	*I believe you must establish a program in person and then I think you can move to a combination. Ideally I would love to see remote and in-person exercise, and when there are issues deal with that in-person, because you can’t do some things at home. I think that all this talk about virtual is really great when you have an ideal situation.* [Provider 6]
	*I think the hybrid would be a better mix. The younger population may want to use different equipment or do different kinds of moves because the exercise right now is geared toward basic moves and the younger people might want to be a bit more maybe adventurous with what kind of exercises they’re doing.* [Provider 4]
	*These tools are complimentary to the clinical assessment. I find that they help inform my decision and guidance if someone’s progression is heading in the wrong direction.* [Provider 3]
Virtual platforms and clinical workflows require further optimization	*One of the challenges with all of these e-tools and platforms is they’re multiple. Everyone has a different preference to their e-communication tool. Some prefer phone, some prefer OTN, some prefer to use Teams or use the App. Sometimes the telecommunication strategy is scheduled but it just doesn’t work.* [Provider 3]
	*If we give them a device then we have to think about do we have the time to really look at what they do all the time?* [Provider 5]
	*If someone is having an exacerbation or difficulties, you can't get a full picture through a text or communication through an administrator. We have to be careful because there needs to be an expectation up front who is monitoring these tools and what would be the response rate. It’s the trend that I'm more interested in because a static value doesn’t tell me the full story.* [Provider 3]
	*I don’t think that our manager appreciates how intense this is for us in terms of staffing. So, it’s not time-saving, there’s always some technical issue, freezing, issues with App notifications, updates to the platform, or someone doesn’t have enough Wi-Fi bandwidth so you need to switch to a phone visit.* [Provider 6]

### Theme 1-Structured exercise benefits both physical and mental health pre-and post-transplant

When working within the confines of their physiology, subject’s participation in structured exercise provided physical benefits to overall conditioning, breathing and muscle strength that increased fitness for surgery and facilitated a quicker post-LTx recovery. A structured program provided clear expectations and goals that supported patients even when they weren’t feeling energetic. In addition to improvements to physical functioning, patient participants felt exercise had a positive impact on their mental resilience and stamina, and that keeping themselves healthy and strong though exercise after LTx was a way to honor their donor.

### Theme 2-CF-specific physiological impairments were a large barrier to exercise

Barriers to exercise focused primarily on physical impairments specific to CF with less emphasis on treatment burden or infection control. Both patient and provider participants described the multi-system presentation of CF as a barrier. In addition to symptoms common to LTx candidates such as dyspnea and fatigue, participants highlighted CF-specific impairments that limited exercise including CF respiratory exacerbations, hemoptysis, digestive issues and hospitalizations. A high daily medical regimen of airway clearance and medications was not identified by patient participants as a large barrier to exercise when at home, but rather exercise was seen as an important treatment supplement that should be part of their structured medical routine. Some participants expressed concern around infection control, however it was felt that during the COVID-19 pandemic the CF population were no longer singled out for specific isolation precautions as all LTx candidates and recipients were required to maintain physical distancing, and there was the option of home exercise.

### Theme 3-Collaborative and supportive relationships, either in-person or virtual, facilitated exercise participation

Patients felt that relationships with the rehabilitation team provided oversight, accountability and allowed a tailored approach that was responsive to changes in their status and addressed other medical conditions such as nutrition and anxiety management to support exercise. This relationship was fostered both in-person and virtually using a remote patient monitoring App in the outpatient setting. In addition, the support of family and friends was very important for ongoing motivation, particularly when they would join the patient during their exercise sessions. Access to home exercise equipment and adequate space in the home was also highlighted as an important facilitator by both patients and providers. Providers felt that a 1 on 1 relationship was necessary to tailor rehabilitation, progress exercise and/or identify a deterioration of function or oxygenation. Providers who worked at the inpatient CF center stressed that their role in rehabilitation differed from the outpatient program. Specifically, their primary aim during admission of an acute CF pulmonary exacerbation was airway clearance, and rehabilitation was focused on the prevention of further deconditioning and loss of muscle mass in order to return them back to their functional baseline rather than increasing endurance and strength from baseline levels.

### Theme 4- More CF-specific exercise evidence, guidance and resources are needed

Patient participants felt that targeted resources and direction would have been helpful during the early stages of their condition to set expectations for lifelong exercise and physical activity. Providers felt that gaps in evidence for exercise remain, particularly in its’ role for airway clearance and in the management of CF-related diabetes. It was felt that in the era of CFTR modulators, exercise may play a larger role in optimizing health-related fitness for people with a potentially different lung health trajectory, longer life expectancy and later referral for LTx.

### Theme 5- Experience with tele-rehabilitation and eHealth tools during the COVID-19 pandemic resulted in preferences for a hybrid model

Both patients and providers shifted rapidly to a tele-rehabilitation model when on-site ambulatory activity was restricted during the COVID-19 pandemic. Although patients reported that they liked the option of home exercise, they also felt that in-person rehabilitation was valuable to ensure safe and effective exercise technique and progression. Overall they preferred some flexibility to mix in-person and on-site sessions, but felt the frequency of in-person sessions should be person-specific. Providers felt that a mix of home and in-person rehabilitation was acceptable when patients had full access to exercise and monitoring equipment, where adherent to home exercise and were not experiencing a major change in their health status. However, providers felt that they could more confidently deal with emerging health changes such as exacerbations and/or increased oxygen requirements in person.

### Theme 6- Virtual platforms and clinical workflows require further optimization

Provider’s virtual care workflows and current eHealth tools impacted outpatient rehabilitation delivery and user experience was varied. Technology issues with connectivity and multiple communication tools that were not integrated with the electronic patient record required workarounds that increased provider workload. The large amount of data that can be collected in an App needs to be available in a summary form and/or show trends over time to be interpreted in a meaningful way that can both inform clinical care and be feasible from a workflow perspective.

## Discussion

There were positive patient and provider perceptions and experiences with rehabilitation before and early after LTx in adults with CF, however further resources and evidence alongside optimization of a tele-rehabilitation or hybrid rehabilitation model is needed.

The finding that both physical and mental benefits arise from structured, regular exercise aligns with previous studies. An international survey of attitudes and experiences of physical activity sent to healthcare providers, parents/caregivers and both pediatric and adult people with CF found physical, psychological and social factors were prime motives for physical activity.^20^ Another online survey in adult LTx recipients with CF more than six months post-transplant found that physical activity was important for physical health and quality of life.^21^ Both surveys found fatigue a common barrier to physical activity, which was also reported from both patient and provider participants in our study.

Virtual relationships with healthcare providers and friends supported participant’s home-based exercise program. The younger age of LTx candidates and recipients with CF may facilitate greater acceptance and usage of ehealth exercise and physical activity tools. Web-based platforms to increase physical activity have been shown to be acceptable and feasible in the CF population, with study participants also expressing preferences for a mobile App interface.^22–24^ As remote monitoring tools, apps and wearables can accumulate significant amounts of data, ensuring appropriate clinical workflows and technical support is essential.^25^ Although patient participants liked the option to exercise at home, there was also a perceived benefit for in-person sessions to safety prescribe and progress exercise. Providers felt they had more ability to assess a change in medical status in person, and some professional groups suggest that tele-rehabilitation may be less suitable for complex populations such as those with severe dyspnea, hypoxemia and recent hospitalizations.^26^ Further clinical and research experience on structure and processes of emerging pulmonary rehabilitation models will inform future practice.

The patient participants in this study were a highly specialized group with CF. In order to be listed for LTx, a person has to have evidence of a support person and must agree to adhere to the medical expectations of the program in terms of mandatory rehabilitation and regular clinic visits with the multidisciplinary LTx team. Three of the five patient participants were awaiting or had undergone re-transplantation. It has been reported that at the time of retransplantation LTx candidates have more renal, cardiac and pulmonary decline compared to their listing for a first transplant.^27^ People listed for retransplantation may have different impairments in skeletal muscle function due to years of corticosteroid and calcineurin inhibitor use which may also impact on the volume of exercise and amount of progression they can tolerate. Their daily medical regimen may also differ in terms of medications, nutritional management and airway clearance therapies compared to when they had not undergone LTx, and thus barriers to exercise may differ.

Lastly, in the age of CFTR modulator therapy, the natural history of CF may continue to change leading to increased life expectancy and a later transition and thus older age at the time of LTx. This has implications for exercise and physical activity counselling and interventions. An increase in age-related co-morbidities such as CF-related diabetes, bone disease, cardiovascular disease and decreases in skeletal muscle mass and function may respond well to regular exercise. As exercise and physical activity is a behaviour, it is important to ensure that cardiorespiratory and musculoskeletal fitness is increased as lung function improves and weight gain occurs with CFTR modulator therapy.^28^

### Limitations

The sample size of patient participants was small and may not have been representative of people with different co-morbidities, support networks and financial backgrounds. This study only examined one large lung transplant program with its’ affiliated adult CF centre and thus the number of potential providers were limited. Over the course of this study the clinical use of CFTR modulator therapy increased at our LTx and CF centers resulting in less people with CF referred or kept on the active waiting list for LTx. This study was also performed during the COVID-19 pandemic when outpatient rehabilitation delivery was undergoing a rapid change towards tele-rehabilitation, on-site supervised activities were restricted and broad infection control mandates were present, which may have impacted perspectives around rehabilitation barriers and facilitators. Recent studies have shown that physical activity levels have been lower in people with CF during the COVID-19 pandemic due to lockdowns, closed facilities and recommended shielding.^29,30^ In addition, as ambulatory outpatient visits were restricted, participants were primarily recruited and consented remotely, which may have excluded some participants who were less confident using technology in this way.

## Conclusion

Structured rehabilitation is perceived as an important component of care pre- and post-LTx, with a preference for a mixed hybrid model that is optimized to meet the needs of both patients and providers. Further evidence and resources to support inpatient, outpatient and home-based rehabilitation in CF is needed.

## Supplemental Material

Supplemental Material - Experiences and perceptions of receiving and prescribing rehabilitation in adults with cystic fibrosis undergoing lung transplantation Click here for additional data file. Supplemental Material for Experiences and perceptions of receiving and prescribing rehabilitation in adults with cystic fibrosis undergoing lung transplantation by Lisa Wickerson, Rajan Grewal, Lianne G Singer and Cecilia Chaparro in Chronic Respiratory Disease
