# Supplementary material for: Experiences and perceptions of receiving and prescribing rehabilitation in adults with cystic fibrosis undergoing lung transplantation
Source: Chron Respir Dis. 2023 Mar 29;20:14799731221139293. doi: 10.1177/14799731221139293 (PMC10064169; doi:10.1177/14799731221139293)
Supplement: Supplemental Material - Experiences and perceptions of receiving and prescribing rehabilitation in adults with cystic fibrosis undergoing lung transplantation [file sj-pdf-1-crd-10.1177_14799731221139293.pdf]

Supplement 1S: Patient and provider interview guides

Patient interview guide

1. **Can you start by telling me about your experience with structured exercise training?**

    - Can you tell me about your experiences with on-site rehabilitation (at UHN or another facility)? What did you like best and least?
    - Can you tell me about your experiences exercising at home? What did you like best and least?
    - Have you been involved in exercise while you were admitted to hospital? What did you like best and least?
    - What is your current exercise participation (weekly amount of exercise done, types of exercise)
    - Do you time your exercise to any particular time of day or around any specific medical therapy?

2. **How does regular exercise affect your health?**

3. **Do you feel you need guidance, education and resources around exercise that is targeted for people who have CF?**

    - What resources have you used?
    - What are the gaps?
    - How do you think exercise is different for people with CF vs. people with other types of lung disease?

4. **What are the barriers in participating in regular exercise?**

    Prompts:

    - *Treatment burden/competing priorities*:

        How much time a day do you spend time daily on:

        - preparing and taking medications (oral, inhaled, nebulized CF meds and/or transplant medications)
        - airway clearance treatments
        - dietary enzymes/vitamins/special diets
        - taking and recording health measures (e.g. blood sugar, weight, spirometry)
        - cleaning medical equipment
        - visits to specialist clinics
        - other

- *Physical health*:
    - How would you rate your overall health?
    - How many times have you been hospitalized in the past year?
    - How has your health changed in the past year?
    - How has your functional level changed in the past year?

- *Social obligations*:
    - Are you involved in paid work or volunteer duties?
    - Are you in school or undertaking continuing education or workplace training?
    - Are you a primary caregiver for a family member?
    - Do you live alone?

- *Environment*:
    - Do you have access to home exercise equipment (if so what types)?
    - Do you have outdoor space near your home to exercise?
    - Do you have access or membership to a shared gym?
    - Do you have infection control concerns about shared gyms or rehabilitation programs?
    - Is a family member concerned about you participating in exercise?
    - Do you prefer to exercise as part of a group or with a workout partner?
    - Do you have a group or workout partner (in-person or online)?

5. **What supports or resources help you to maintain an exercise program?**

I want to ask you about E-health tools or technology such as vital sign monitors, activity trackers, phone health apps or web-based applications that interpret health data.

6. **Do you have any experience with e-health tools/technology?**

    - Do you have access to a smart phone or tablet and reliable Wi-Fi/ high speed internet?
    - Which tools have you used?
    - Why have you used these tools?
    - How do you feel they support your exercise participation?
    - How have you shared this generated health data with your UHN team?
    - How would you like to share this generated health data with your UHN team?

7. **How could the rehabilitation program at UHN be optimized and delivered to best meet your needs?**

    - What would be a preferred rehab model (on-site, home or a mix of on-site and remote)?
    - How could on-site sessions complement home exercise sessions?

- What would be the ideal frequency and length of exercise sessions?
- What should the UHN rehab program keep doing?
- Is there anything specific you would like added?

Is there anything I haven't asked about that you would like to tell me?

Provider interview guide

1. **Could you start by telling me about the setting you work in and how you are involved in the rehabilitation of CF patients?**
   - How many years have you worked in this area?
   - Do you work with people both before and after lung transplant?
   - Could you expand on how you prescribe, monitor and/or counsel exercise for CF patients? What do you tell them? How do you tell them? (e.g. handouts, verbal, written)
   - Could you describe any strategies that have been particularly effective? Is there room for improvement?

2. **What are the benefits of exercise training for lung transplant candidates and recipients with CF?**

3. **Do you feel you there is sufficient CF-specific evidence and resources around exercise?**

4. **Describe barriers patients experience when it comes to regular exercise participation.**

   What is the impact of?

   - Treatment burden/competing priorities
   - Physical health
   - Social obligations (family, work/volunteer, education, household)
   - Environmental- infection control issues, social support, access to equipment
   - How do these barriers differ compared to people with other lung diseases?

5. **What do you think would facilitate increased adherence and motivation for exercise?**

6. **Can you describe your communication with the other members of the healthcare team (inside your own organization and externally) with respects to the rehabilitation of CF patients?**

    a. What are the specific challenges with communication?
    b. Are there different issues with communication pre-transplant and post-transplant?
    c. Are there different issues with communication when they are admitted to hospital?

I want to ask you about E-health tools or technology such as vital sign monitors, activity trackers, phone health apps or web-based applications that interpret health data.

7. **Do you have any experience using e-health tools in the rehabilitation of CF patients?**

    - What kinds of tools have you used?
    - Why did you choose to use these tools?
    - Is tele-rehabilitation or remote monitoring part of your clinical program? If yes, how is it used (e.g. assessment, treatment, education)?
    - How do you think you could implement e-health tools into regular clinical practice?
    - (*For UHN providers only*) Have you used the Vivify platform at UHN? Describe what you like best and least about this platform?
    - Have you used other virtual platforms (if yes please list)? What do you like best and least about the platform?
    - Were there any clinical guidelines you used or followed to guide your virtual practice?
    - How does virtual practice and e-health tools affect your clinical workflow?

8. **Are there any e-health tools that your patients know of or currently use?**

9. **What challenges or barriers have you faced in virtual practice or with e-health tools?**
    - How did you address these challenges?
    - How did you address patient barriers?
    - Were there factors that prevented you from providing tele-rehabilitation?
        - Patient?
        - Program?
        - Environmental?
        - Technological?

10. **Please describe your ideal rehabilitation program for CF patients before and after lung transplant.**

- Do you have a preference for an on-site vs. home vs. hybrid (on-site and home) program?
- How could on-site sessions complement home exercise sessions?
- What would be the ideal frequency and length of exercise sessions?
- What should the UHN rehab program keep doing?
- How can exercise during hospital admission be optimized pre-transplant?
- What CF-specific modifications would you make?
- What e-health tools would be most helpful in your daily practice?
- What results would you like to get from an e-health tool?
- How would these results be interpreted and used in your clinical decision-making?

Is there anything else you feel I should know about your experience providing rehabilitation care to people with CF??

Supplement 2S: Patient satisfaction survey administered through the App

Pre-Transplant App Patient Satisfaction Survey[a]

Strongly Agree/ Agree/ Neither Agree or Disagree/ Disagree/ Strongly Disagree

I am comfortable using the App

The App empowers me to manage my health condition

The health tips and education library in the App help me learn about my health condition

I feel the App helps my preparation for surgery

I can easily find information I need to prepare for surgery

The App will be helpful in managing my care at home during my recovery from surgery

Virtual care features (e.g. videoconferencing, texting, education library, nutritional guidelines, symptom surveys, health tips) will be helpful for my self-recovery at home

Post-Transplant App Patient Satisfaction Survey[a]

Strongly Agree/ Agree/ Neither Agree or Disagree/ Disagree/ Strongly Disagree

The App empowers me to manage my health condition

The App improves my level of confidence in taking care of myself at home, after surgery

I can easily find information I need to help me recover from surgery

Video conferences are helpful for my self-recovery at home

Texting with my care team is helpful for my self-recovery at home

Daily check in surveys are helpful for my self-recovery at home

Education health tips are helpful for my self-recovery at home

I felt this App helped me avoid unnecessary emergency room visits

Do you feel that there was good communication about your care between doctors, nurses and other hospital staff?

Did you receive enough information from hospital staff about what to do if you were worried about your condition or treatment after you left the hospital?

Overall I am satisfied with using the App to support my journey through recovery

[a] Developed by a lung transplant clinical working group and MyCareConnection project management team at the University Health Network. The one-time survey that is send pre-

transplant after 14 days following registration on the App and post-transplant three months following surgery
